# Case series of rhinocerebral mucormycosis occurring in diabetic patients 

**Published:** 2015

**Authors:** Rasoul Mohammadi, Mohsen Meidani, Kamyar Mostafavizadeh, Bijan Iraj, Pooria Hamedani, Sayed Mohammad Amin Sayedain, Mojgan Mokhtari

**Affiliations:** 1 Department of Medical Parasitology and Mycology, School of Medicine, Isfahan University of Medical Sciences, Isfahan, Iran.; 2 Department of Infectious Diseases, Al-Zahra Hospital, Isfahan University of Medical Sciences, Isfahan, Iran.; 3 Department of Endocrinology, Isfahan University of Medical Sciences, Isfahan, Iran.; 4 Department of Cardiology, Chamran Hospital (Heart Center), Isfahan University of Medical Sciences, Isfahan, Iran.; 5 Department of Pathology, School of Medicine, Isfahan University of Medical Sciences, Isfahan, Iran.

**Keywords:** Rhinocerebral mucormycosis, Diabetes Mellitus, Iran

## Abstract

**Background:**

Rhinocerebral mucormycosis is a fatal infection typically affecting diabetic or immunosuppressed patients. In most cases, infection is caused by inhalation of fungal spores. Mortality rate of patients is very high (40-85%).

**Case Presentation:**

In this study, three diabetic patients with rhinocerebral mucormycosis were presented. The etiologic agents of mucormycosis in two patients were isolated and identified by sequence analysis and data were registered in Gene bank database.

**Conclusion:**

In patients with mucoreosis, early detection, surgical excision and appropriate debridement, suitable antifungal therapy, and control of risk factors like diabetes mellitus are the main parameters of successful management of this lethal infection.

**R**hinocerebral mucormycosis is an acute and often lethal opportunistic fungal infection typically affecting diabetic (50% of the cases) or immunocompromised patients caused by fungi of the class zygomycetes (1-3). Recent series have described a mortality of approximately 40% in diabetics with rhinocerebral mucormycosis. The infection has high incidence in diabetic patients due to the greater availability of glucose to the pathogen, lower response of T-cells, reduced serum inhibitory activity against the Rhizopus in lower pH, and increased expression of some host receptors that mediate the invasion of human epithelial cells through microorganism (4-6). The aim of this study was to present patients with rhinocerebral mucormycosis who were successfully treated due to well-timed diagnosis of infection, and identification of etiologic agent by molecular method. 

## Case Reports

**Case 1: **A 27-year-old woman was admitted to the emergency department of Al-Zahra Hospital, Isfahan, Iran, who had nausea, vomiting, weakness, loss of consciousness (LOC), and dysarthria for injury due to blunt. Biochemical tests and arterial blood gas (ABG) analysis provided information on the following: (BS=957 mg/dl, Cr=3 mg/dl, pH=6.8, HCO3=2.7). The patient was hospitalized for diabetic ketoacidosis (DKA), and intravenous (IV) insulin infusion therapy (0.1 U/kg/h) and intravenous hydration were administered. By reason of LOC, endotracheal intubation was used. After 3 days, mouth examination showed necrotic lesions (2.5×2.7 cm) in the left soft palate.

**Figure 1:**
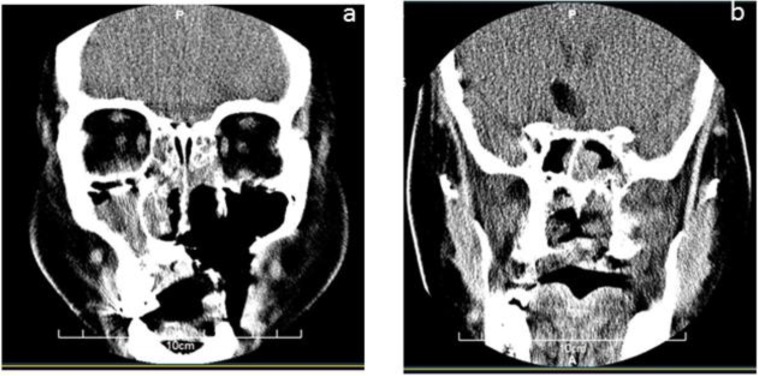
a: there is evidence of bone destruction and surgical resection in right maxillary sinus and right side palate; b: CT scan of face and paranasal sinuses, there is evidence of air fluid level in both maxillary sinus compatible with acute sinusitis. Mucosal thickening is also seen in ethmoidal and sphenoid sinuses

CT scan of the paranasal sinuses (PNS) was performed. There was evidence of air fluid level in both maxillary sinuses compatible with acute sinusitis. Mucosal thickening was also seen in ethmoidal and sphenoid sinuses (figure 1). Brain magnetic resonance imaging (MRI) findings approved CT scan results, and pansinusitis and bilateral mastoiditis were seen, but there was no invasion to the brain vessels. Amphotericin B (1 mg/kg/day) was administered, and debridement of sinuses, ethmoidectomy, bilateral antrotomy, and resection of soft palate was performed using a video endoscopy. Histopathological findings and the results of culture confirmed our diagnosis. The isolate was identified as *Rhizopus oryzae* by sequence analysis (Gene Bank accession number is KJ577257). The dosage of amphotericin B changed to (1.5 mg/kg/day) and then to (3 mg/kg/day), and after two weeks, the patient was discharged from the hospital. After 4 months, there was no evidence of recrurrence of infection.

**Figure 2:**
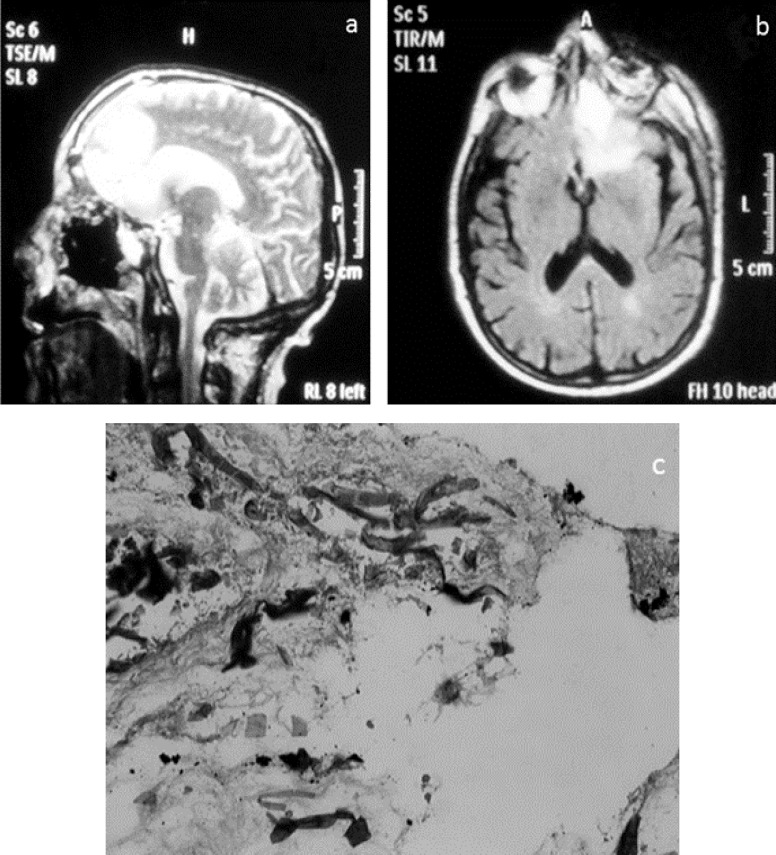
a and b: the brain MRI study with sagittal and coronal plane. There is a signal change in the left frontal lobe as high signal intensity on T2 FLAIR with edema and minimal mass effect, assymetry of the left orbit compared to the right side. Small vessel ischemia and maxillo-ethmoidal sinusitis are noticed; c: ribbon like and aseptate hyphae in necrotic tissue (H&E stain, ×40)

**Case 2: **A 59-year-old man who had persistent headache and nasal discharge was admitted to the emergency department of Al-Zahra Hospital. After a few days, left eye discharge appeared. He had nineteen years history of diabetes (diabetes mellitus type 2), hypertension, and kidney malfunction. Brain MRI study showed an abscess in the left orbit and sinusitis in ethmoid and maxillary sinuses. There was edema and a signal change in the left frontal lobe (Figure 2). Histopathological findings from biopsy of maxillary sinus presented aseptate hyphae in necrotic tissue (Figure 2). The culture result was positive. We identified the isolate using sequence analysis as *Rhizopus oryzae* (Gene Bank accession number is KF228585). Debridement of sphenoidal sinus and necrotic tissues was performed and amphotericin B (1 mg/kg/day) was prescribed for him. Antifungal therapy changed to posaconazole (PCZ) (5 mg/kg) due to renal failure of patient. Left eye was enucleated. Antifungal therapy with posaconazole (5 mg/kg) was continued and the patient was released from the hospital after two weeks. We followed-up the patient for 4 years, and fortunately, there was no recurrence of infection.

**Figure 3:**
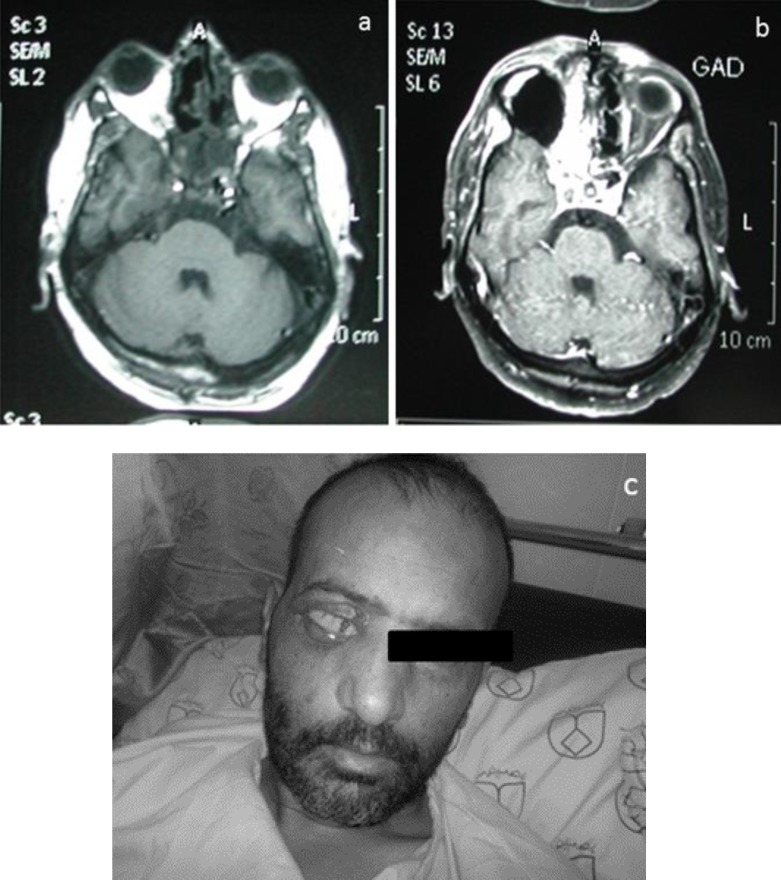
3a: In MRI study, there is evidence of enhanceable soft tissues density in right ethmoidal air cell with bone destruction and extension to right and retro-orbit and intracranial area. Opascification of right sphenoidal and ethmoidal sinus also is seen; 3b and 3c: right orbit is enucleated

**Case 3: **A 45-year-old man had black nasal discharge, ptosis of right eye, bulging on right side of his face, and facial pain. Biochemical tests indicated data on the following fasting blood sugar (FBS): 370 mg/dl, hemoglobin A1c: 11%). The patient was hospitalized for intravenous (IV) insulin infusion therapy (0.1 U/kg/h). Brain MRI study with multiplanar images revealed only a few periventricular and deep white matter hyperintense foci. These were compatible with ischemic changes. Grey matter signal, cerebral ventricles, major intracranial vessels, basal ganglia and brain stem were normal. Opacification of paranasal sinuses and nasal cavity was seen due to sinusitis, mostly on the right side with extension to right orbit. Due to involvement of fundus of right orbit and optic nerve, infection due to fungus origin was suggested. Fortunately, invasion to brain vessels was not seen. Cochleate-amphotericin B (1 mg/kg/day) was prescribed for him and maxillary and ethmoid sinuses were debrided. A biopsy specimen from maxillary sinus was taken. Hematoxylin and eosin stain displayed broad aseptate hyphae with right angles in the background of necrotic debris, but in this case, the isolate did not grow on the synthetic media. In consequence of orbit involvement, right eye enucleation was carried out. After 40 days, posaconazole (5 mg/kg) was added to his antifungal regimen, and the patient was discharged after 15 days (figure 3).

## Discussion

There are some predisposing factors for mucormycosis such as hematological malignancies, severe burns, neutropenia, diabetes mellitus, and the use of corticosteroids (7). Definitive diagnosis should be made by clinical manifestation of the disease, histopathological examination of infected tissues, culture (culture studies are usually unsuccessful), and radiographic features. Mortality rate of mucormycosis is high in spite of antifungal therapy. Nonetheless, all cases of this study were treated in consequence of good management of the infection. Javadi et al. (8) reported 9 mucormycosis cases among diabetic patients in Tehran. All patients were treated with AmB, and 7 out of 9 patients died. Fata et al. (9) introduced 22 patients who were treated with AmB. The mortality rate was 75% in their study. These findings highlighted the role of posaconazole in treatment of mucormycosis. The use of cochleate amphotericin B (CAmB) to treat mucormycosis, may not be desirable due to the nephrotoxicity effects of drug (10). Unfortunately, liposomal amphotericin B (LAmB) was not accessible and so we treated the patients with amphotericin B.

In Conclusion, Early detection, surgical excision and appropriate debridement, suitable antifungal therapy, and control of risk factors like diabetes mellitus are the main parameters of successful management of this lethal infection among diabetic patients.
